# Selection criteria for linear regression models to estimate individual tree biomasses in the Atlantic Rain Forest, Brazil

**DOI:** 10.1186/s13021-018-0112-6

**Published:** 2018-12-07

**Authors:** Carlos Roberto Sanquetta, Ana Paula Dalla Corte, Alexandre Behling, Luani Rosa de Oliveira Piva, Sylvio Péllico Netto, Aurélio Lourenço Rodrigues, Mateus Niroh Inoue Sanquetta

**Affiliations:** 1 Forest Science Department, Federal University of Paraná, Curitiba, Brazil; 2 Graduate Programme in Forestry, Federal University of Paraná, Curitiba, Brazil

**Keywords:** Equation fitting, Modeling, Regression, Tropical forest, Woody species

## Abstract

**Background:**

Biomass models are useful for several purposes, especially for quantifying carbon stocks and dynamics in forests. Selecting appropriate equations from a fitted model is a process which can involves several criteria, some widely used and others used to a lesser extent. This study analyzes six selection criteria for models fitted to six sets of individual biomass collected from woody indigenous species of the Tropical Atlantic Rain Forest in Brazil. Six models were examined and the respective fitted equations evaluated by the residual sum of squares, adjusted coefficient of determination, absolute and relative estimates of the standard error of estimate, and Akaike and Schwartz (Bayesian) information criteria. The aim of this study was to analyze the numeric behavior of these model selection criteria and discuss the ease of interpretation of them. The importance of residual analysis in model selection is stressed.

**Results:**

The adjusted coefficient of determination ($R^{2}_{adj.}$) and the standard error of estimate in percentage (*Syx*%) are relative model selection criteria and are not affected by sample size and scale of the response variable. The sum of squared residuals (*SSR*), the absolute standard error of estimate (*Syx*), the Akaike information criterion and the Schwartz information criterion, in turn, depend on these quantities. The best fit model was always the same within a given data set regardless the model selection criteria considered (except for *SSR* in two cases), indicating they tend to converge to a common result. However, such criteria are not always closely related across different data sets. General model selection criteria are indicative of the average goodness of fit, but do not capture bias and outlier effects. Graphical residual analysis is a useful tool to this detection and must always be used in model selection.

**Conclusions:**

It is concluded that the criteria for model selection tend to lead to a common result, regardless their mathematical formulation and statistical significance. Relative measures of goodness of fitting are easier to interpret than the absolute ones. Careful graphical residual analysis must always be used to confirm the performance of the models.

## Background

There are different methods of calculating biomass and carbon storage in forests. Usually these methods combine information from forest inventories and expansion factors or fitting linear regression models [1]. Biomass models, usually fitted by linear regression (called allometric equations by some authors) can be used to obtain indirect estimates, by using tree measurement data (such as dbh, height, among others) coming from forest inventory, and are widely used for this purpose. Soares and Tomé [2] advocate the use of biomass equations, because the architecture of trees changes over time and under prevailing site conditions, altering the fixed proportion implicit in the expansion factors. Equations for biomass estimation require the examination of different models, which must be judged by some statistical indicators of goodness of fit. Selecting the best model, in principle, is a simple task, since there are well known criteria for this purpose. Many tools for the choice of the “best model” have been suggested in the literature [3]. However, different objectives in modeling can exist besides prediction, which require an integrated vision of the different model selection criteria.

Model selection has occupied the minds of many researchers, and a large number of publications devoted to this subject can be found in the literature [4–12]. Particularly in biomass estimation this issue is not deeply explored. Although model selection criteria for biomass estimation are widely used, a specific discussion on their significance and application has not been yet published.

Criteria for model selection must incorporate goodness of fit and parsimony, allowing that several models examined can be simultaneously compared [13]. Among the selection criteria most commonly adopted are the following: adjusted coefficient of determination, maximum likelihood test, Akaike information criterion, Akaike information criterion not biased to small samples, and Schwarz information criterion (also called Bayesian) [13]. There are variations of the mathematical formulations of these criteria in the literature, though their rationale are similar.

*R*^2^ (coefficient of determination) is perhaps the measure of fitting most widely used in linear regression modeling, but, according to some authors, it has been improperly used [14]. After the Anscombe’s publication on *R*^2^ [15], various criticisms have been made about the use of it as a model selection criterion. The author’s analysis has become famous, when he proposed a consideration on four series of different data that resulted in the same value of *R*^2^ in the fitting of the straight-line model, the so-called “Anscombe’s quartet”. Kvalseth [14] has discussed the several potential pitfalls in using the *R*^2^ inadvertently. Some authors consider this measure as antiquated and with many restrictions [5, 11, 16].

One of *R*^2^ features is that the increase in the number of parameters causes a concomitant increase of its value, giving the false impression that a certain model is better than another. Another point is that models with different numbers of coefficients cannot be compared directly by *R*^2^. Therefore, the adjusted *R*^2^ should be used instead [17]. Other statistical analyses traditionally employed are the size of the absolute (*Syx*) and the relative (*Syx*%) error of estimate, and the graphical residual analysis as well. Vanclay [18] suggests analyzing the data graphically, noting also the F-values of the regression and the statistic prediction sum of squares (PRESS) [12, 19].

The information criteria proposed by Akaike [20] and Schwarz [21] have been used and recommended for model selection. These alternative indices would be a better combination of ability to detect the goodness of fit and, therefore, the quality of the model, as well as penalize complex models that could mask the selection results.

Although this matter is of great importance for biomass and carbon modeling of woody species, we have not found in the literature research papers devoted to the compare the results obtained with a variety of data sets, by using analyzing different criteria for selecting models. In this work, the behavior of six selection criteria are evaluated to estimate individual biomass through six linear regression models fitted to actual data of different woody species indigenous of the Tropical Atlantic Rain Forest in southern/southeastern Brazil.

The aim of this study was to analyze the behavior of six model selection criteria, typically used to judge the goodness of fit of the resulting equations fitted to six different data series with wide biomass range. Besides the sample size and response variable size on these criteria, we also examined the numeric relations between them. We discuss the ease of interpretation of the model selection criteria and stress the importance of the graphical residual analysis to detect bias in estimates.

## Methods

### Data sources

Six sets of dry biomass data were used in this study, totaling 330 individuals of various woody species indigenous in the Tropical Atlantic Rain Forest in southern/southeastern Brazil (Table 1). Data sets 1–3 are composed of aboveground biomass measures (trunk + branches + foliage), whereas series 4–6 data come from total biomass (aboveground + belowground) measurements. Biomass was measured through destructive method (simple separation of compartments) [22], which consisted of weighing fresh biomass in the field and further analysis in the laboratory to obtain the oven dry biomass.

**Table 1:** Data source for fitting linear regression models to biomass estimation of different woody species indigenous of the Tropical Atlantic Rain Forest, Brazil

Data set	n	Location UTM (m)	Mean ± S.D. of biomass	CV (%)	SE (%)	95% confidence interval
1. *M. skvortzovii* Sendulsky bamboo growing in natural forest^a^	30	General Carneiro, Paraná State 457.589–467.617 7.085.594–7.075.828	0.26 ± 0.13	49.63	18.53	0.21–0.31
2. *M. skvortzovii* Sendulsky bamboo growing in natural forest^b^	30		260.59 ± 129.32	49.53	18.53	212.30–308.87
3. Native mixed-species natural stand^a^	30		1493.24 ± 1449.87	97.10	36.26	951.84–2034.63
4. Mixed-species restoration plantation (complete series)^c^	180	Seropédica, Rio de Janeiro State 637.586–640.342 7.484.977–7.483.459	16.95 ± 23.87	70.02	26.14	12.51–21.38
5. Mixed-species restoration plantation (reduced series with outliers)^c^	30		59.64 ± 32.36	54.25	20.26	47.56–71.73
6. Mixed-species restoration plantation^c^ (reduced series without outliers)	30		43.40 ± 21.96	50.60	18.90	35.20–51.60

CV (%) = coefficient of variation, SE (%) = sampling error. 95% confidence interval

^a^Aboveground biomass in kg

^b^Aboveground biomass in g

^c^Aboveground + belowground biomass in kg

Data sets of plants with broad range of diameter at breast height (1.30 m from the ground level) and of total heights were taken and deliberately utilized. Individual biomass averages ranging from 0.26 kg (*Merostachys skvortzovii* bamboo) up to 1493 kg (indigenous old-growth tree species in mixed-species natural stand). All data sets had 30 plants, except for one of them (native species in restoration forest plantations, data set 4) with 180 plants. The data sets 5 and 6 are subsets of the 4th series, with a smaller number of cases, without and with outliers, respectively. 2.2 data analysis.

The dependent (response) variable in the regression models was *w* (oven dry biomass) and the independent (input) variables were *dbh* (diameter at breast height or 1.3 m above the ground—cm) and *h* (total height—m), and combinations of both, as seen below:

The models examined in this study were:

1 $$ w_{i} = \beta_{0} + \beta_{1} \,(dbh^{2} h)_{i} + e_{i} $$

2 $$ w_{i} = \beta_{0} + \beta_{1} \,(dbh^{2} )_{i} + e_{i} $$

3 $$ w_{i} = \beta_{0} + \beta_{1} \,(dbh)_{i} + e_{i} $$

4 $$ \ln \,(w_{i} ) = \beta_{0} + \beta_{1} \,(\ln (dbh)_{i} ) + \beta_{2} \,(\ln (h)_{i} ) + e_{i} $$

5 $$ \ln \,(w_{i} ) = \beta_{0} + \beta_{1} \ln \,(dbh^{2} h)_{i} + e_{i} $$

6 $$ \ln \,(w_{i} ) = \beta_{0} + \beta_{1} \,(\ln \,(dbh)_{i} ) + \beta_{2} \,(\ln \,(h)_{i} ) + \beta_{3} \,(dbh^{2} h)_{i} + e_{i} $$

where *β*_0_, *β*_1_, *β*_2_ are the coefficients to be determined, *ln* is logarithm neperian, *e*_*i*_ is random error.

The models examined include formulations with 2, 3 and 4 coefficients. The purpose of this variation was to evaluate the effect of model’s complexity on the behavior of the model selection criteria. The equations obtained after fitting were evaluated regarding the model selection criteria (Table 2) and graphical residual analysis. The statistical significance of each coefficient was examined by means of the t-test. The following hypotheses were formulated: If H_0_ (β_j_ = 0) is not rejected, then *x*_*j*_ (independent variable) should be removed from the model, because this variable has not influenced on the response of *w* in a meaningful way. If H_0_ (β_j_ = 0) cannot be accepted, then *x*_*j*_ contributes significantly to explain the responses of *w*.

**Table 2:** Statistical criteria for model selection applied to biomass estimation of different woody species indigenous of the Tropical Atlantic Rain Forest, Brazil

	Criterion	Formula	
1	Sum of squares of the residuals	$SSR = \sum\limits_{i - 1}^{n} {e_{i}^{2} } = \sum\limits_{i - 1}^{n} {(w_{i} - \hat{w}_{i} )^{2} }$	(7)
2	Adjusted coefficient of determination	$R^{2}_{adj.} = 1 - \frac{(n - 1)}{(n - p)}(1 - R^{2} )$ where $R^{2} = 1 - \frac{{\sum\nolimits_{i - 1}^{n} {e_{i}^{2} } }}{{\sum\nolimits_{i - 1}^{n} {(w_{i} - \bar{w})^{2} } }}$	(8)
3	Relative standard error of the estimate	$Syx\% = \frac{syx}{{\bar{y}}}100$ where $Syx = \sqrt {\frac{{\sum\nolimits_{i - 1}^{n} {e_{i}^{2} } }}{n - p}}$	(10)
4	Akaike information criterion [20]	$AIC = - 2n\left( {\frac{ - n}{2}\ln \left( {\frac{1}{n}\sum\limits_{i - 1}^{n} {e_{i}^{2} } } \right)} \right) + 2p$	(11)
5	Akaike information criterion not biased for small samples^a^, when (n/p) < 40	$AIC_{c} = - 2n\left( {\frac{ - n}{2}\ln \left( {\frac{1}{n}\sum\limits_{i - 1}^{n} {e_{i}^{2} } } \right)} \right) + 2p\frac{n}{(n - p - 1)}$	(12)
6	Schwartz’s information criterion [21]	$BIC = - 2n\left( {\frac{ - n}{2}\ln \left( {\frac{1}{n}\sum\limits_{i - 1}^{n} {e_{i}^{2} } } \right)} \right) + \ln (n)p$	(13)
7	Residuals (in %)	$r_{i} = \frac{{(w_{i} - \hat{w}_{i} )}}{{w_{i} }}100$	(14)

$\hat{w}_{i}$ = estimated biomass. *w*_*i*_ = actual biomass. In *AIC*, *AICc* and *BICp* must be increased by 1, which refers to one degree of freedom for variance

^a^According to [11]. Where *n* = number of data; *p* = number of parameters of the model (number of coefficients including the intercept + 1)

The equation fitting was carried out by means of the ordinary least squares method. For the logarithmic models, the values were transformed back to the original response variable to calculate the statistical model selection criteria. In these cases, the logarithmic bias (discrepancy) was corrected by the Meyer’s factor (*MF*):

15 $$ MF = e^{{0.5\,Syx^{2} }} . $$

The detection of influential points in the fitting (outliers) was performed by means of DFFITS and COOK [23, 24] distance values. Normality and variance homogeneity were evaluated by the Shapiro–Wilk and White tests, respectively.

## Results and discussion

### Results

**Fig. 1:**
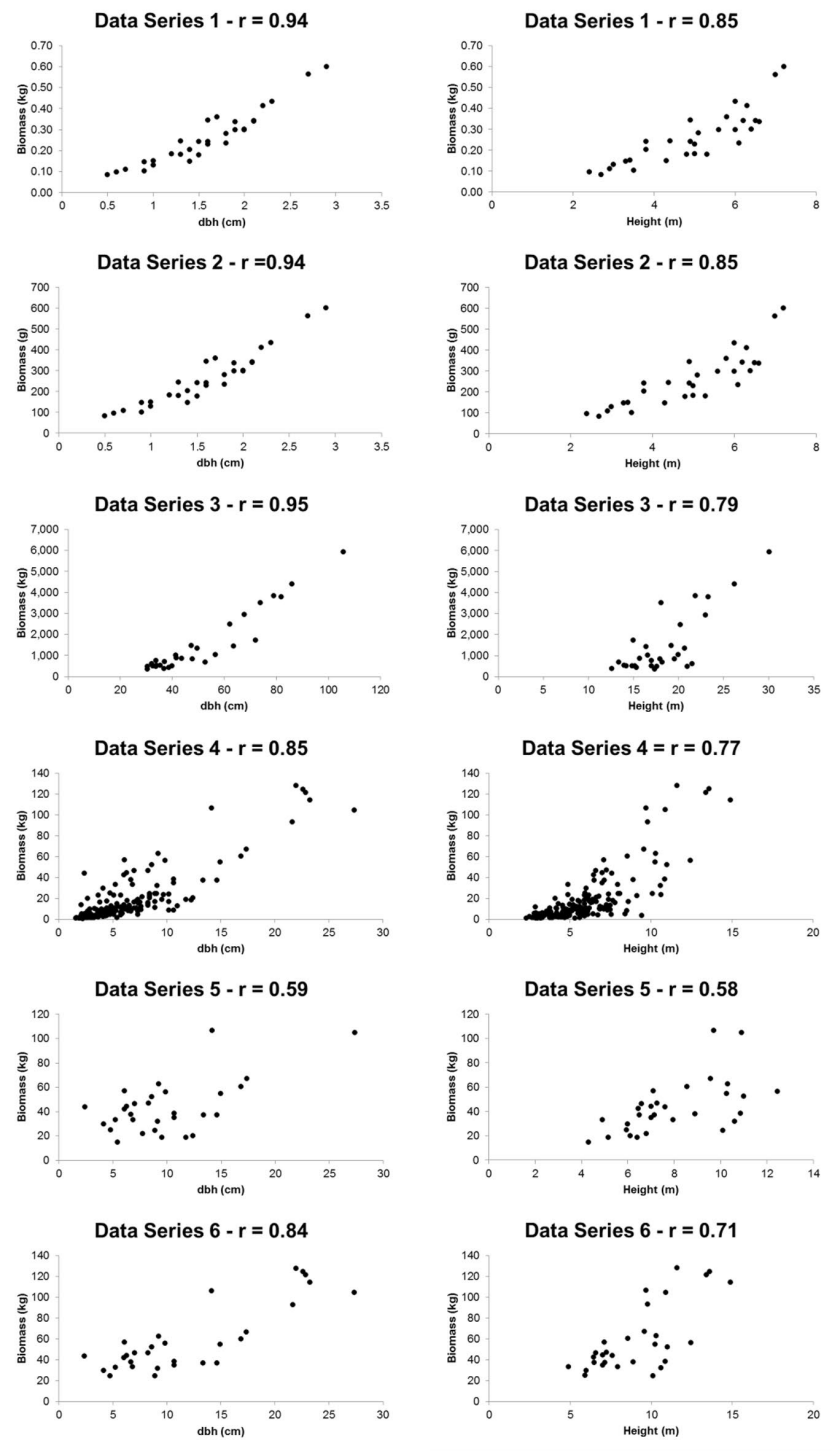
Relationship between *dbh* and biomass in six data series (1–6, as shown in Table 1) for different forest tree species indigenous in the Atlantic Rain Forest, Brazil. Series numbers are shown in Table 1

The relationships between *dbh* and height, and the respective biomasses, were positive, as expected, with a greater or lesser degree of dispersion, depending on the data series. The correlation of biomass with *dbh* was greater than with height, as measured by the Pearson´s coefficient (Fig. 1). All the linear correlations between biomass and *dbh* and *h* were statistically significant, so that these two measures can be properly used as input variables in biomass modeling. Some coefficients of the equations were not statistically significant (p < 0.01), indicating that the respective models could be reduced in number of parameters (Table 3). However, in order to keep consistency and avoid unnecessary complexity, we decided to maintain the original formulas throughout the analysis.

**Table 3:** Coefficients of linear regression models to biomass estimation of woody species indigenous to the Atlantic Rain Forest, Brazil

Data set	Coeff.	Model 1	Model 2	Model 3	Model 4	Model 5	Model 6
1. *M. skvortzovii* growing in natural forest (biomass in kg)	*β*_0_	0.121974	0.080625	− 0.067196	− 2.045500	− 2.440900	− 1.963500
	*β*_1_	0.008520	0.063605	0.207457	1.057200	0.421600	0.854700
	*β*_2_	–	–	–	0.115176	–	0.011440
	*β*_3_	–	–	–	–	–	0.009587 ns
2. *M. skvortzovii* growing in natural forest (biomass in g)	*β*_0_	− 67.195600	80.625200	121.974400	4.862200	4.466800	4.944300
	*β*_1_	207.457000	63.605500	8.519700	1.057200	0.421600	0.854700
	*β*_2_	–	–	–	0.115176	–	0.011440
	*β*_3_	–	–	–	–	–	0.009587 ns
3. Native mixed-species natural stand	*β*_0_	269.734200 ns	− 190.405000 ns 0.404900	− 0.020012	− 3.187200	− 3.034100	− 4.353000
	*β*_1_	0.020000	0.570500	69.121800	1.830400	0.939800	2.013600
	*β*_2_	–	–	–	1.058400	–	1.247900
	*β*_3_	–	–	–	–	–	− 0.000001 ns
4. Native mixed-species restoration plantation (complete series)	*β*_0_	8.697100	5.597000	− 11.402400	− 1.390800	− 1.049100	− 1.332100
	*β*_1_	0.016510	0.197855	4.601500	1.051500	0.647783	1.018300
	*β*_2_	–	–	–	1.084300	–	1.073600
	*β*_3_	–	–	–	–	–	0.000027 ns
5. Native mixed-species restoration plantation (reduced series without *outliers*)	*β*_0_	32.601900	31.343100	17.806700 ns	1.235600 ns	2.339400	2.038792
	*β*_1_	0.009945	0.098619	2.590100	0.120959 ns	0.206500	− 0.189838 ns
	*β*_2_	–	–	–	1.058000	–	0.915639
	*β*_3_	–	–	–	–	–	0.000150 ns
6. Native mixed restoration plantations (reduced series with *outliers*)	*β*_0_	36.326800	33.347300	11.784300 ns	1.622700	2.118800	2.926700
	*β*_1_	0.011285	0.137935	3.967500	0.421100	0.270000	0.060223 ns
	*β*_2_	–	–	–	0.593400 ns	–	0.289279 ns
	*β*_3_	–	–	–	–	–	0.000129

In general, the fittings for the data sets 1–3 could be considered satisfactory regarding $R^{2}_{adj.}$ and *Syx*%. However, loss of accuracy for the fitted models to data set 3, as indicated by the higher value of *Syx*% in spite of the high $R^{2}_{adj.}$ (Table 4), was evidenced. We noticed a remarkable reduction of $R^{2}_{adj.}$ and increase of *Syx*% for data sets 4–6 in comparison to the previous ones. For the data sets 4 and 5, model fittings could be considered satisfactory if $R^{2}_{adj.}$ figures alone are taken into account, but in the case of data set 6 they could not. Considering only *Syx*%, model fitting to data set 4 could not be acceptable, whereas those to data set 4 and 5 could be regarded as fair. From these analysis we can say that $R^{2}_{adj.} \times Syx\;\%$ relationship is not always as clear as expected and that model selection criteria are affected in different ways, depending on the data features and the model examined. Thus, decision making should not be done based on only one measure.

**Table 4:** Criteria for selecting linear regression models to biomass estimation of some woody species inigenous in the Atlantic Rain Forest, Brazil

Data set	Model	*SSR*	$R^{2}_{adj.}$	*Syx*	*Syx*%	*AIC*	*BIC*
1. *M. skvortzovii* growing in natural forest (biomass in kg)	1	0.048772 (3)	0.8958 (3)	0.0417 (3)	16.02 (3)	− 185.73 (3)	− 182.45 (3)
	2	0.038193 (1)	0.9184 (1)	0.0369 (1)	14.17 (1)	− 193.07 (1)	− 189.79 (1)
	3	0.055108 (4)	0.8823 (4)	0.0444 (4)	17.02 (4)	− 182.07 (4)	− 178.79 (4)
	4	0.057474 (5)	0.8727 (5)	0.0461 (5)	17.71 (5)	− 178.13 (5)	− 174.12 (5)
	5	0.063102 (6)	0.8652 (6)	0.0475 (6)	18.22 (6)	− 178.00 (6)	− 174.72 (6)
	6	0.039924 (2)	0.9147 (2)	0.0378 (2)	14.49 (2)	− 191.74 (2)	− 188.46 (2)
2. *M.* *skvortzovii* growing in natural forest (biomass in g)	1	48,772 (3)	0.8958 (3)	41.74 (3)	16.02 (3)	228.73 (3)	232.02 (3)
	2	38,193 (1)	0.9184 (1)	36.93 (1)	14.17 (1)	221.40 (1)	224.68 (1)
	3	55,108 (4)	0.8823 (4)	44.36 (4)	17.02 (4)	232.40 (4)	235.68 (4)
	4	57,474 (5)	0.8727 (5)	46.14 (5)	17.71 (5)	236.34 (5)	240.34 (5)
	5	63,102 (6)	0.8652 (6)	47.47 (2)	18.22 (6)	236.46 (6)	239.74 (6)
	6	39,924 (2)	0.9147 (2)	37.76 (6)	14.49 (2)	222.73 (2)	226.01 (2)
3. Native mixed-species natural stand	1	5,422,069 (3)	0.9079 (3)	440.05 (3)	29.47 (3)	370.07 (3)	373.35 (3)
	2	4,300,629 (2)	0.9269 (2)	391.91 (2)	26.25 (2)	363.35 (2)	366.40 (2)
	3	6,385,976 (5)	0.8915 (5)	477.57 (5)	31.98 (5)	374.98 (5)	378.26 (5)
	4	6,702,675 (6)	0.8819 (6)	498.24 (6)	33.37 (6)	379.10 (6)	383.11 (6)
	5	6,002,299 (4)	0.8980 (4)	463.00 (4)	31.01 (4)	373.12 (4)	376.40 (4)
	6	3,228,136 (1)	0.9452 (1)	339.54 (1)	22.74 (1)	354.51 (1)	357.79 (1)
4. Native mixed restoration plantation (complete series)	1	24,955 (4)	0.7539 (4)	11.84 (4)	69.87 (4)	891.74 (4)	898.12 (4)
	2	25,745 (5)	0.7461 (5)	12.03 (5)	70.96 (5)	897.34 (5)	903.73 (5)
	3	28,006 (6)	0.7238 (6)	12.54 (6)	74.02 (6)	912.50 (6)	918.89 (6)
	4	19,337 (1)	0.8082 (1)	10.45 (1)	61.67 (1)	847.82 (1)	857.40 (1)
	5	21,009 (2)	0.7928 (2)	10.86 (2)	64.11 (2)	860.76 (2)	867.14 (2)
	6	24,333 (3)	0.7601 (3)	11.69 (3)	68.99 (3)	887.19 (3)	893.58 (3)
5. Native mixed-species restoration plantation (Reduced series without *outliers*)	1	7307 (2)	0.7778 (1)	15.25 (1)	25.58 (1)	168.35 (1)	171.63 (1)
	2	8274 (3)	0.7219 (3)	17.06 (3)	28.61 (3)	175.07 (3)	178.36 (3)
	3	9034 (6)	0.6912 (4)	17.98 (4)	30.15 (4)	178.22 (4)	181.50 (4)
	4	8402 (4)	0.6556 (5)	19.33 (5)	32.41 (5)	178.27 (5)	182.07 (5)
	5	8950 (5)	0.6492 (6)	19.51 (6)	32.70 (6)	177.19 (6)	180.33 (6)
	6	7070 (1)	0.7363 (2)	16.62 (2)	27.86 (2)	173.48 (2)	176.76 (2)
6. Native mixed-species restoration plantation (reduced series with *outliers*)	1	6516 (1)	0.4590 (2)	16.15 (2)	37.22 (2)	171.79 (2)	175.07 (2)
	2	8154 (3)	0.3874 (3)	17.19 (3)	39.61 (3)	175.51 (3)	178.79 (3)
	3	9054 (4)	0.3312 (6)	17.96 (6)	41.38 (6)	178.15 (5)	181.43 (4)
	4	9713 (5)	0.3549 (4)	17.64 (4)	40.64 (4)	178.65 (6)	182.66 (6)
	5	10,274 (6)	0.3374 (5)	17.88 (5)	41.19 (5)	177.87 (4)	181.15 (5)
	6	7732 (2)	0.4766 (1)	15.89 (1)	36.61 (1)	170.79 (1)	174.07 (1)

Number in parenthesis represent the ranking for the best fitting models

*SSR* sum of squared residuals, $R^{2}_{adj.}$ adjusted coefficient of determination, *Syx* residual standard deviation, *Syx*% residual standard deviation in percentage, *AIC* Akaike information criterion, *BIC* Schwartz’s information criterion

The best fit equations to data sets 1 and 2 was model 2, considering all the model selection criteria, i.e., lowest *SSR*, *Syx*, *Syx*%, *AIC* and *BIC* values of and largest $R^{2}_{adj.}$. Equation (6) was the best for data sets 3, 5 and 6, and model 1 gave the best results for data set 4. The model selection criteria did to not affect the best fit model decision, except for *SSR* which gave distinct results for data sets 5 and 6. Therefore, the best fit model does not change and it does not matter which criterion is being used to rank the goodness of fitting.

**Fig. 2:**
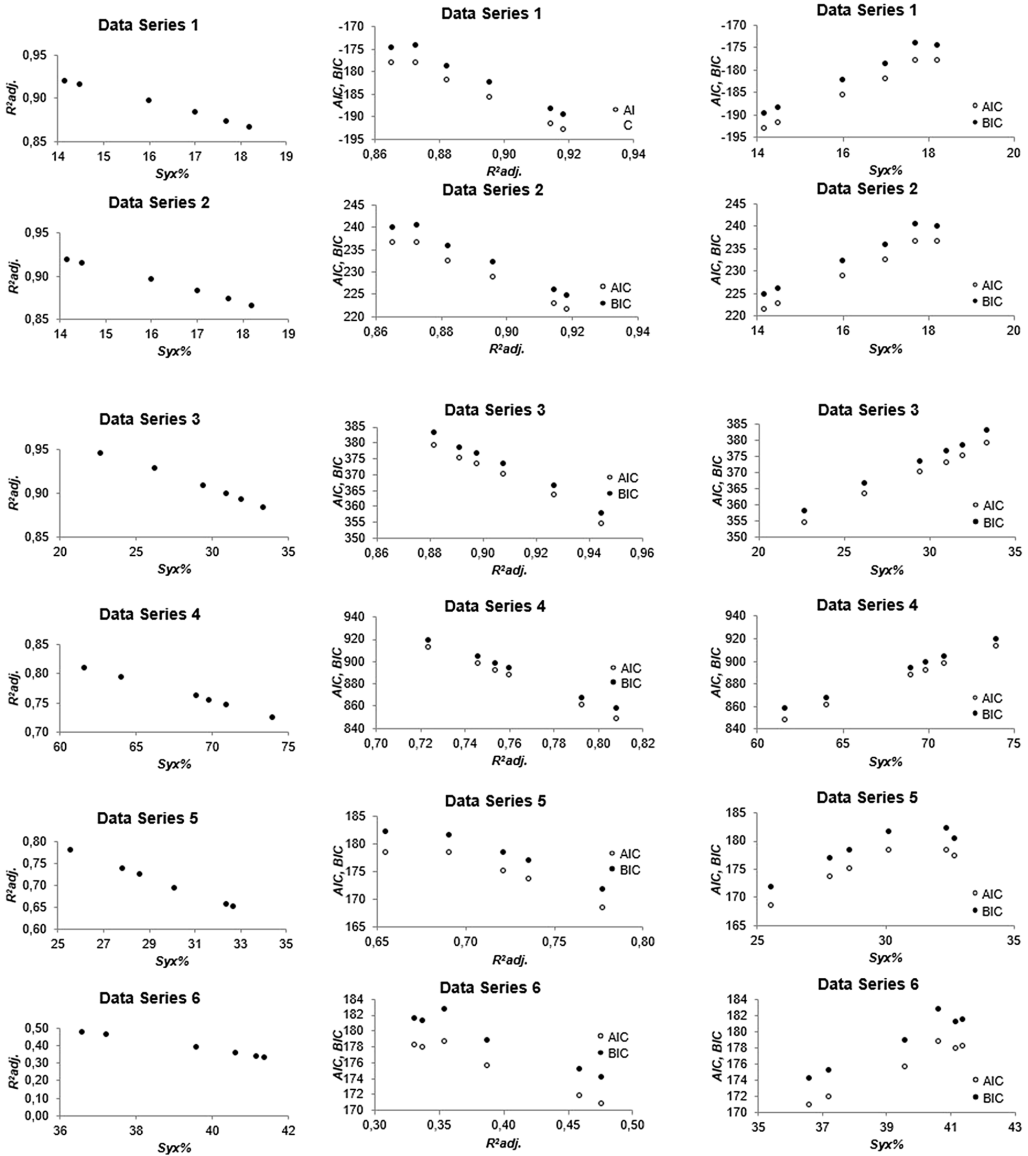
Relationship between selection model criteria for biomass estimation of woody species indigenous in the Atlantic Rain Forest, Brazil

This work revealed a close relationship between the general model selection criteria within each data series, since they are all calculated on the basis of the square difference of actual and predicted values, the *SSR*. Relations among them tend to be linear for all combinations of selection criteria, though some deviations from linearity regarding *AIC* and *BIC* were noticed (Fig. 2). The relations were direct, i.e., the larger *SSR* the larger the values of the selection criteria *Syx*, *Syx*%, *AIC* and *BIC*, and reverse for $R^{2}_{adj.}$. From this analysis, it can be said that all selection criteria converge to a common result within the same data set.

The *SSR* values are closely related to the size of the response variable, considering that it is an absolute measure of the quadratic difference of the actual and estimated values. The same can be said in relation to *Syx*. Note that not only the effect of the unit of measure on data sets 1 and 2 appears on the values of these model selection criteria. *AIC* and *BIC* are transformed absolute measures of fitting and assume somewhat different behaviors. In the case of data sets 1 and 2, negative values are noticed for the first and positive for the second, suggesting that these measures do not only suffer the effect of the unit of the response variable. It is important to note that the *AIC* and *BIC* values do not imply to any change in the ranking of the goodness of fit of the models, and hence no practical advantage in using them for this purpose was evidenced in this study.

**Fig. 3:**
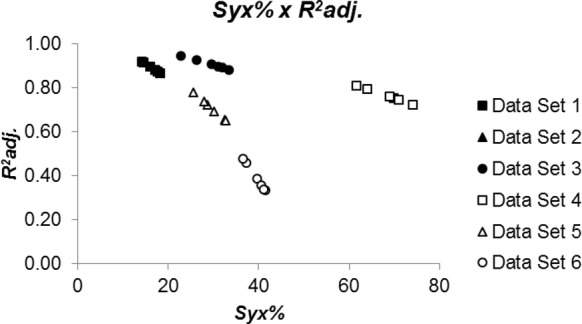
Relationship standard error of estimate and coefficient of determination of linear regression models fitted to biomass estimation of woody species indigenous in the Atlantic Rain Forest, Brazil

The close relationship of the selection criteria did not apply when the data sets are analyzed altogether, even for the relative measures, i.e., $R^{2}_{adj.}$ and *Syx*% (Fig. 3). As mentioned before, we detected that fitted biomass equations with high $R^{2}_{adj.}$ may result in high *Syx*% (e.g. data set 4), what is not expected. Similarly, low $R^{2}_{adj.}$ may be followed by relatively low *Syx*% (e.g. data set 6). It means that even those relative straightforward and easy-to-understand criteria widely used in model selection may fail in decision making. Caution should be taken when using any of these model selection criteria.

**Fig. 4:**
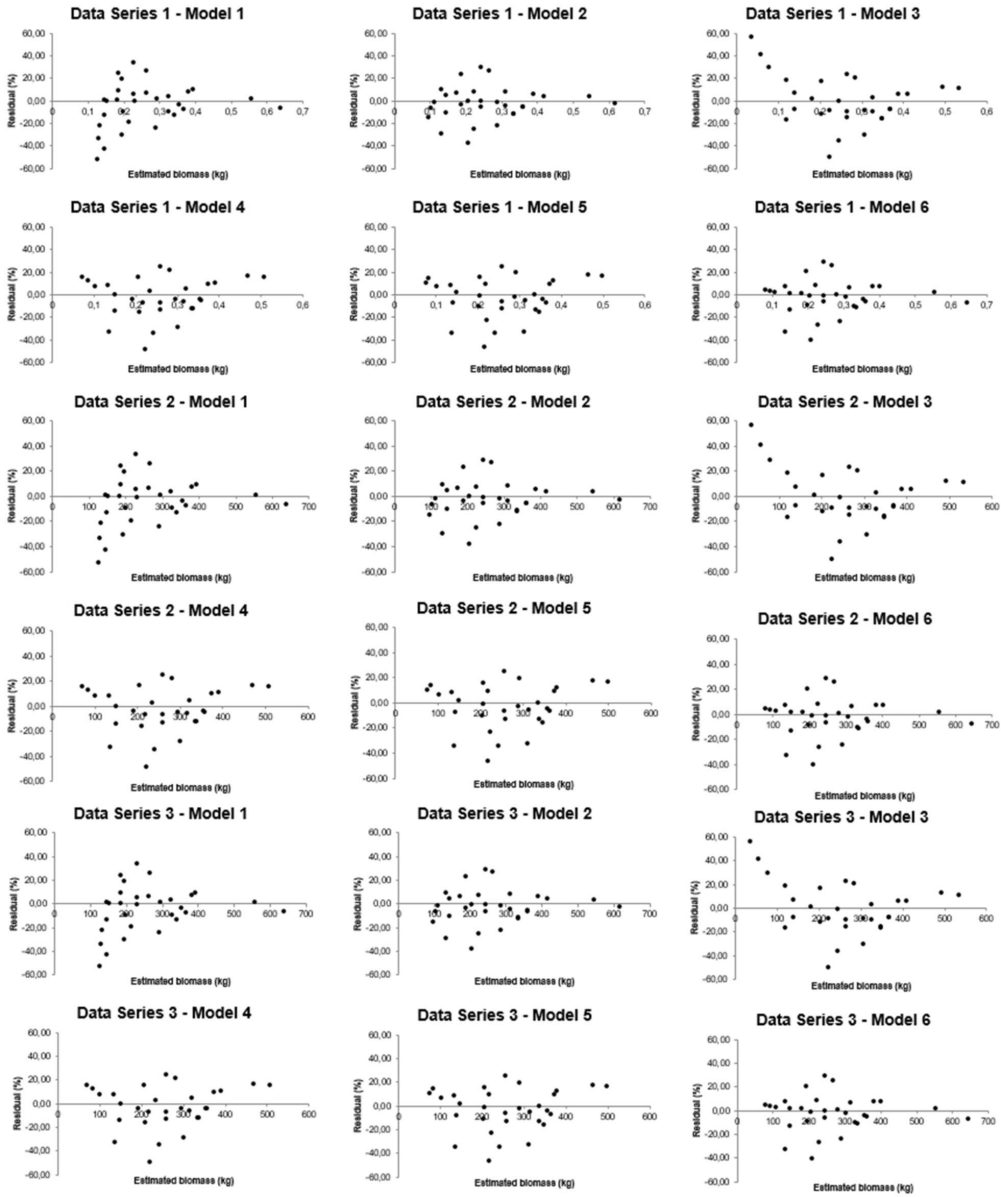
Residual analysis for linear regression models fitted to biomass estimation of woody species indigenous in the Atlantic Rain Forest, Brazil

**Fig. 5:**
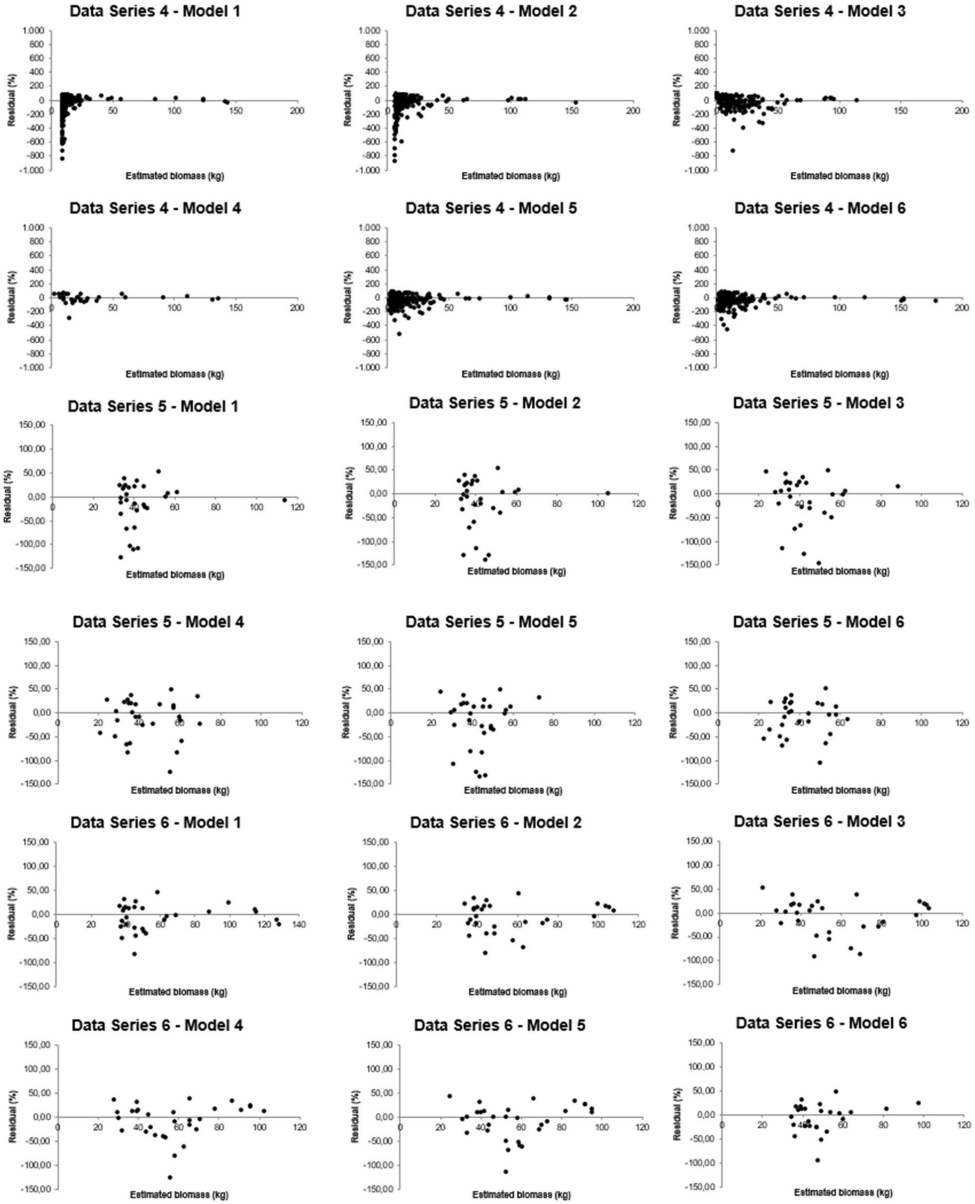
Residual analysis for linear regression models fitted to biomass estimation of woody species indigenous in the Atlantic Rain Forest, Brazil

Residual graphical analysis performed on all the models and data sets used on this study revealed important particularities of the fittings that were no apparent from the other model selection criteria (Figs. 4, 5). All the equations presented good general fitting criteria for data set 1, which could lead one to believe that any of these models would be reliable. However, graphical analysis detected the presence of bias in the residual distribution in some cases [e.g. Eqs. (1) and (3)]. Equation (1), for instance, did not present normality in the statistical test. The same applies to data set 2.

Biases were also evidenced in model fittings for data set 3. Equation (1), for example, that showed acceptable behavior by the general model selection criteria, gives biased biomass estimates and lack of normality of residues. Residual analysis revealed strong biases in data set 4 biomass prediction, particularly for the small-sized individual estimates generated by the fitting of Eqs. (1) and (3). This was not detected by the general model selection criteria. All the models examined were negatively affected by lack of normality and heteroscedasticity of residuals. In other words, all the equations fitted to this data set, in principle, should be rejected.

In addition, biased estimates were also noticed for Eqs. (1)–(3) fitted to data set 5. Though model was considered the best fit to this data set by the model selection criteria, but from the residual analysis another model should be chosen. Finally, residual analysis showed that all fitted models provide overestimation of the large-sized individuals of data set 6. However, the range of the residuals of the models fitted to this data set, which showed poor model selection indicators, indicated that the estimates are not as bad as one can suppose from the other model selection criteria.

Then, invisible facts from the model selection criteria could be revealed by the residual analysis, which can be useful either in detecting biases and/or showing the width of the residuals individual by individual, which is not possible by the general model selection criteria.

### Discussion

Many distinct models have been proposed and several model selection indicators used in biomass estimation. Perhaps, in some cases the modelers and users do not care about the quality and reliability of such models. However, superficial analysis of the general model selection criteria may lead to critical errors.

Some model selection criteria are particular interesting and useful. Interpretation of $R^{2}_{adj.}$ and *Syx*% values is straightforward and allows us to understand whether the fitting is good or not, while the other criteria sometimes are not so friendly. This does not necessarily mean that these are ideal criteria for model selection and that are free of possible misleading interpretations, as shown here and emphasized also by the literature.

$R^{2}_{adj.}$ and *Syx*% are not affected by the magnitude of the response variable, once they are relative measures. In turn, *SSR* and *Syx* vary with biomass unit in a direct way, i.e., these statistics are directly affected by the dimension of the dependent variable. *AIC* and *BIC* values are also affected by the size of the dependent variable, but the changes in values do not maintain a direct relationship with the magnitude of the dependent variable. It happens because such information criteria are logarithmic transformations of *SSR*. It was observed that when the biomass values are in kg, the corresponding *AIC* and *BIC* are negative and when in grams become positive. *AIC* cannot be used to compare models tested for different sets of data [11]. The same can be said to *BIC*. Moreover, they cannot be used to compare models fitted for the same data set but with different units of the response variable. This should be taken into account in model selection.

Some absolute model selection measures (e.g. *AIC* and *BIC*) may not be sensitive to the existence of outliers. This indicates that these measures may not be sensitive enough to capture the effect of such abnormal data on model fitting. Outliers are not uncommon in modeling forest biomass and impossibility of detecting outliers is very problematic. This was one of the arguments against the *R*^2^ in Anscombe’s [15] work and by other authors who criticized this criterion.

It is fundamental at this point to highlight the importance of the residual analysis on the selection of regression models for plant biomass estimation. This analysis is very helpful in verifying the presence of bias in model fitting. Taking data set 4 as an example we are able to realize serious biased estimation of small-sized individuals, which was not evidenced from another manner (Figs. 4, 5). Although general criteria can be very helpful for model selection, the presence of outliers and bias in estimates can only be detected through the residual analysis. Residual analysis can used to evidence whether a model is adequate and/or help to discriminate the best fit when various models are fitted to the same data set.

Model selection are related one each other. This is conditional to the formulation of the information criteria examined. If it is assumed here that the parameters of the model can be estimated by the maximum likelihood method in ordinary linear regression models [13, 25]:

8 $$ \ln \left[ {L\left( {\hat{\theta }_{p} \left| y \right.} \right)} \right] = \left( {\frac{ - n}{2}\ln \left( {\frac{1}{n}\sum\limits_{i - 1}^{n} {e_{i}^{2} } } \right)} \right) $$

where $\ln \left[ {L\left( {\hat{\theta }_{p} \left| y \right.} \right)} \right]$ is the maximum likelihood for the parameters of the model.

Assuming this relationship, the close practical relationship between the information criteria and $R^{2}_{adj.}$ can be readily noticed, in spite of the theoretical difference among them (Fig. 2).

The literature is prolific in works criticizing the use of the coefficient of determination as a useful criterion for selecting models. Figueiredo Filho et al. [26] claim that there is no substantive significance in the use of *R*^2^ as indicative of adjustment of a model. Many researchers have abandoned completely the use of the coefficient of determination, mainly after the publication by Anscombe [15].

Several authors have presented alternatives, making apology to a criterion and criticism to others. According to Vismara [16], criteria have been sought to assess the best model by approximation to describe data, among several possibilities, with different functional relations and with different numbers of parameters. The author describes the advantages of using the *AIC* and suggests that it could be an excellent tool for selecting empirical models for predictions in the forest environment.

Burnham and Anderson [11], in turn, point out that *AIC* represents a new paradigm in the selection of models from empirical data and that the model selection based on the so-called “information theory” represents a quite different approach in the statistical science in comparison to the usual hypothesis tests.

Despite the favorable or unfavorable positions of the several authors to one or another criterion, it is evident that the criteria present similarities in their practical applications, in spite of differences in their mathematical formulations and the theoretical basis behind them. This study shows that $R^{2}_{adj.}$ and *AIC* are related one each other. No clear practical advantage of using *AIC* or *BIC* in model selection was evidenced in this research. *AIC* and *BIC* are tremendously affected by the size of the data set in use, which makes it more difficult to use the approach in a broader and more generic analysis of model fitting Although *R*^2^, according to the literature, presents many limitations for use in model selection [5], the other criteria may show similar pitfalls.

Model selection criteria are general indicators of the behavior of the theoretical model against empirical data. They tend to give a good indication of the goodness of fit to the extent that the data have a regular pattern, i.e., without great dispersion and outliers, and that logical models are tested against the actual data. It is also important to point out that in regression modeling, as in any other sampling scheme, it is definitely important to use an amount of data that is representative of the real world. Perhaps the great sin of Anscombe’s work has been to force an illogical adjustment of the model to a database consisting of only 11 values, and with outliers. The problem is in the data set itself and not in *R*^2^. The database and the philosophy behind model fitting are more relevant in this sense.

On the other hand, the great merit of Anscombe’s work was to highlight the importance of graphical data analysis before performing any model fitting. In this context, the graphical analysis of the residuals should be considered as the tool to help the modeler to select one among the various tested models. The importance of the residuals analysis is widely addressed by Dubbelman [27] and Cook and Weisberg [24]. Just looking at the $$ R^{2}_{adj.} , $$ it can be concluded that the fittings made to the data set 4 could be good (at least reasonable), but when we observe the distribution of residuals is evident the weakness of the predictions. By observing the values of *AIC* and *BIC* one could inadvertently conclude that there is not much difference of fitting for data sets 5 and 6. It would not be possible to identify the presence of outliers in the series 6.

$$ R^{2}_{adj.} , $$ taking the criterion of Theil (1961), is based on the assumption that one of the specified models is correct. In this case, if $$ \hat{\sigma }_{j}^{2} = \frac{{SQR_{j} }}{{(n - k)_{j} }} $$ is the estimate *σ*^2^ of the jth model, then $$ E(\hat{\sigma }_{j}^{2} ) = \sigma^{2} $$ for the correct model, but is ≥ *σ*^2^ for the model poorly specified. According to Maddala [28], a model that has all the explanatory variables of the correct model, but also a number of irrelevant variables will result in $$ (\hat{\sigma }_{j}^{2} ) = \sigma^{2} . $$ Thus, the choice of the model based on *σ*^2^ minimum leads on the average to choose the correct model [29]. How to minimize *σ*^2^ means maximize $$ R_{adj.}^{2} , $$ therefore, the best model is the one with the highest $$ R^{2}_{adj.} , $$ i.e., the rule of $$ R^{2}_{adj.} $$ maximum.

Maddala and Lahiri [29] indicate that the main problem with this rule is that the model that has all the explanatory variables of the correct model, but also a number of irrelevant variables will also result in $E(\hat{\sigma }_{j}^{2} ) = \sigma^{2} .$ Thus, only taking this rule does not allow you to choose the correct model. Ebbeler [30] discussed regarding this aspect, concluding that the probability of choosing the correct model is considerably smaller than 1, when another model includes a number of irrelevant variables. The effect of omission of important variables or inclusion of irrelevant variables is widely discussed by Gujarati [17] and Greene [31]. We found that the F-test of the analysis of variance for the equation informs the statistical significance of the adjusted equation, which is at the same time a measure of the statistical significance of *R*^2^. According to Gujarati [17], the F-test is given by:$F = \frac{SSE/(k - 1)}{SSR(n - k)} = \left( {\frac{n - k}{k - 1}} \right)\left( {\frac{SSE}{SST - SSE}} \right) = \left( {\frac{n - k}{k - 1}} \right)\left( {\frac{SSE/SST}{1 - (SSE/SST)}} \right),$ being $R = \frac{SSE}{SST},$ the value of *F* can be calculated by: $F = \left( {\frac{n - k}{k - 1}} \right)\left( {\frac{{R^{2} }}{{1 - R^{2} }}} \right) = \left( {\frac{{R^{2} /(k - 1)}}{{(1 - R^{2} )/(n - k)}}} \right),$ being *SSE* the sum of squares explained and *SST* the total sum of squares. The assumptions made for the statistical test are the same as those proposed for the *F* test. The *F* test is a comprehensive test of the equation and in the majority of cases taken into account as a criterion in the choice of an equation; therefore this only reinforces the notion that the value of *R*^2^ should not be simply dismissed as a criterion in the choice of an equation.

The literature on model selection has brought to light a number of statistical tests that can be performed for this purpose. There is not ideal criterion for model selection, especially for tree biomass. This depends on the objectives of the modeling and of the data you have at hand [5, 32]. Therefore, it is essential that in model fitting, particularly for biomass of woody plants, that certain basic steps should be followed, namely: (1) Make a broad exploratory data analysis; (2) Study the behavior of variables and their trends; (3) Select appropriate models to be tested, which should describe the relations of cause and effect between the variables, even if empirically made; (4) Use the various selection criteria for models to achieve the best choice, particularly the graphical analysis of residuals; (5) Use the fitted equations with parsimony, avoiding to extrapolate their estimation ability.

It was evidenced that no statistical test, alone, has been able to indicate the equation to be used. Even when the overall tests were combined, they ended up running into difficulties especially when evaluated the individual tests for the coefficients. In addition, even when analyzed together, comprehensive test and individual test, in some cases, the selected equation could not meet some of the assumptions tested for validation of the classic model of linear regression. This indicates that the choice of equations must pass through three stages. The authors suggest, in this work, to start with the evaluation of the assumptions of linear regression, followed by the analysis of individual coefficients (significance of the coefficients and standard deviation) and the assessment of the overall quality of the adjustment (taking a series of statistics) and finally to perform the residual analysis, in order to find the best specifications for the model.

If the main concern of the linear regression analysis is only the statistical inference on the coefficient estimates, to explore the method of least squares would be good enough. However, linear regression analysis involves the inference about the equality between the estimators and a population sample. For this reason, it should be verified which are the delineated hypotheses for a classical linear regression model, which are addressed in detail by Gujarati [17], Greene [31] and Wooldridge [33].

In general, it is not usually assumed, when modeling biomass, that the statistical model to be fitted to data is in the first moment known, so that the only issue to be addressed in modeling would be the estimation of the coefficients. Thus, the choice of models for biomass is performed after the statistical analysis of the adjustments. Usually the first evaluation is made on the statistics’ overall quality of the equation. However, it was verified that these do not take into account some basic assumptions of the linear regression model, for example: average random error equal to zero, homoscedasticity of errors, absence of autocorrelation between the errors, proper specification of the regression model and absence of multicollinearity. The heteroscedasticity and autocorrelation depend on particular values of explanatory variables in the sample [29]. These two constraints are easy to be violated, especially when modeling forest biomass; the reasons for doing so are obvious. What is expected of the residuals in an equation is that they should behave with the same properties as the real errors, i.e., the errors should have zero mean, constant variance and be serially independent; residuals also should assume these properties.

One of the hypotheses of the classic model of linear regression is that the errors $\hat{e}_{i}$ in equation have common variance *σ*^2^, being this hypothesis known as homoscedasticity. When the errors do not have constant variance they present heteroscedasticity. One way to detect heteroscedasticity is to build a graph of predicted residuals to check whether there is any systematic pattern in the distribution of residuals that suggests the heteroscedasticity of the errors [29]. Moreover, statistical tests to check for heteroscedasticity are available, as example, the test proposed by White [34], which involves the regression in all explanatory variables, their squares and cross-products.

The main consequences of heteroscedasticity in estimators of least squares are that they do not present bias, but they are inefficient and the main problem is that the estimates of the variances are skewed, invalidating, as a result, the tests of significance. Maddala [29] presents the proof of these two hypotheses. Therefore, a fundamental review to be conducted at a first moment in the selection of models for biomass is to evaluate the homoscedasticity. Therefore, a fundamental review to be conducted at a first moment in the selection of models for biomass is to evaluate the homoscedasticity. For cases of detection of heteroscedasticity in forest biomass, the solution to this problem would be to turn the series in logs.

Another assumption of the classical linear regression model is the absence of multicollinearity—term used by Frisch [35], i.e., it implies that two or more independent variables should not be correlated linearly between themselves. If they are, then not all parameters are estimable. In the case of modeling biomass, this is a hypothesis hardly likely to be violated, since the independent variables used are not linearly correlated because, in most cases, they can be combined variables (the example of *dbh*^2^*h*). However, if we still want to check, an appropriate test would be the inflation factor of the variance. Maddala [29] has discussed at length about this hypothesis of the classic linear regression model.

An important hypothesis that must be evaluated in linear regression modeling is whether errors are or not normally distributed. A good way to test this hypothesis is to use the Shapiro–Wilk test, widely discussed by Huang and Bolch [36]. Commonly, when we are modeling tree’s biomass this problem will appear, due to the nature of the data. One of the ways suggested by Maddala [29] is to escape from not normality, i.e., transform the data so that the assumption of normality will remain valid. One of many possible ways to make an asymmetric distribution become symmetric is to raise y to a power or apply the log. Tukey [37] covers in detail the processing of data. The author suggests that the changes help to make the model approximately linear, errors more homoscedastic and normally distributed. The author shows a great family of transformations, as well as later did Box and Cox [38]. For Box and Watson [39], studying the robustness of the tests of regression coefficients, when the errors are not normal, they argue that the empirical distribution of the explanatory variable *x* is approximately normal, the usual tests will hold the significance levels assumed.

In view of these facts, it is suggested that the evaluation of the modeling of biomass should start by two basic assumptions of the model classic linear regression: homoscedasticity and normality. The individual analysis of the coefficients is a good technique to start the evaluation of equations after this process, because it makes no sense to keep in the model coefficients that are not statistically significant. As a result, the choice of the equation must pass by the statistics of the overall quality of the adjustment and the conclusion made after a deep analysis of the residuals.

## Conclusions

1. The model selection criteria ($$ R^{2}_{adj.} , $$ *Syx*, *Syx*%, *AIC* and *BIC*) are useful as general indicative of goodness of fit;
2. These criteria keep relations among them within the same data set, because they are based on the root mean square of the difference between the actual and predicted values;
3. No practice advantage of the use of *AIC* and *BIC* in comparison to the adjusted coefficient of determination, despite the eloquent defense of these information criteria by various authors and the criticism to the traditional *R*^2^;
4. The model selection criteria may fail in not detecting biases and other special data and fitting features that are only possible through the examination of residuals;
5. In biomass modeling, it is recommended to perform a detailed exploratory data analysis, a pre-selection of logical models to be tested and use several model selection criteria, including necessarily a careful residual analysis.

## Abbreviations

$R^{2}_{adj.}$: adjusted coefficient of determination
Syx: absolute estimates of the standard error
Syx%: relative estimates of the standard error
*w*: dry biomass
*dbh*: diameter at breast height or 1.3 m above the ground
*h*: total height in meters
AIC: Akaike information criterion
AICc: Akaike information criterion not biased for small samples
BICp: Schwartz’s information criterion or Bayesian
MF: Meyer’s factor
